# Translocation of Distinct Alpha Synuclein Species from the Nucleus to Neuronal Processes during Neuronal Differentiation

**DOI:** 10.3390/biom12081108

**Published:** 2022-08-12

**Authors:** Katharina Pieger, Verena Schmitt, Carina Gauer, Nadja Gießl, Iryna Prots, Beate Winner, Jürgen Winkler, Johann Helmut Brandstätter, Wei Xiang

**Affiliations:** 1 Department of Biology, Animal Physiology/Neurobiology, Friedrich-Alexander-Universität Erlangen-Nürnberg, 91058 Erlangen, Germany; 2 Department of Molecular Neurology, University Hospital Erlangen, Friedrich-Alexander-Universität Erlangen-Nürnberg, 91054 Erlangen, Germany; 3 Department of Stem Cell Biology, University Hospital Erlangen, Friedrich-Alexander-Universität Erlangen-Nürnberg, 91054 Erlangen, Germany

**Keywords:** alpha synuclein, Parkinson’s disease, *SNCA* duplication, nuclear alpha synuclein

## Abstract

Alpha synuclein (aSyn) and its aggregation are crucial for the neurodegeneration of Parkinson’s disease (PD). aSyn was initially described in the nucleus and presynaptic nerve terminals. However, the biology of nuclear aSyn and the link of aSyn between subcellular compartments are less understood. Current knowledge suggests the existence of various aSyn species with distinct structural and biochemical properties. Here, we identified a C-terminal-targeting aSyn antibody (Nu-aSyn-C), which has a high immunoaffinity towards aSyn in the nucleus. Comparing the Nu-aSyn-C antibody to aSyn antibodies developed against phosphorylated or aggregated forms, we observed that nuclear aSyn differs from cytosolic aSyn by an increased phosphorylation and assembly level in proliferating cells. Employing Nu-aSyn-C, we characterized aSyn distribution during neuronal differentiation in midbrain dopaminergic neurons (mDANs) derived from human-induced pluripotent stem cells (hiPSCs) and Lund human mesencephalic cells, and in primary rat hippocampal neurons. We detected a specific translocation pattern of aSyn during neuronal differentiation from the nucleus to the soma and finally to neuronal processes. Interestingly, a remarkable shift of Nu-aSyn-C-positive species towards neurites was detected in hiPSC mDANs from a PD patient carrying aSyn gene duplication. Together, our results reveal distinct nuclear and cytosolic aSyn species that redistribute during neuronal differentiation—a process that is altered in PD-derived neurons.

## 1. Introduction

**Figure 1:**
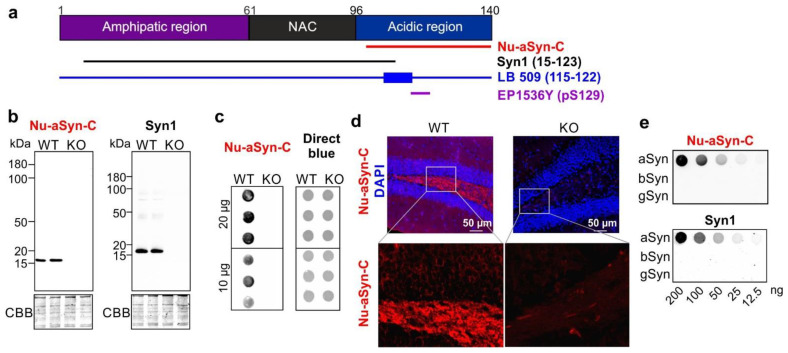
Nu-aSyn-C antibody is specific to aSyn. (**a**) Structure of human aSyn and antibodies against different aSyn epitopes applied in this study. (**b**) Denaturing WB analysis of brain lysates of WT and aSyn KO mice using Nu-aSyn-C and Syn1 antibodies demonstrates specificity of both antibodies to aSyn in WT mice. (**c**) Non-denaturing dot blot analysis of WT and aSyn KO mouse brains reveals immunosignals in WT mice only. An amount of 10–20 µg of total protein extract was loaded for WB (**b**) or dot blot (**c**). Loading of the total protein extract was controlled by CBB (WB, (**b**)) or using direct blue 71 dye (dot blot, (**c**)). (**d**) IHC analysis of WT and KO brain sections using the Nu-aSyn-C antibody shows specific immunosignals in WT brains only (example images from the hippocampus in d; example images from the cortex in Figure S1. (**e**) Dot blot analysis of human recombinant a-, b-, and gSyn using Nu-aSyn-C and Syn1 antibodies. Both antibodies do not cross-react with b- and gSyn.

Alpha synuclein (aSyn) is a small protein with 140 amino acid residues, which is highly abundant in the human brain [1,2]. It belongs to the synuclein family consisting of three distinct members: aSyn, beta Syn (bSyn), and gamma Syn (gSyn). The primary structure of aSyn can be divided into three core regions: the amphipathic N-terminus (amino acids: 1–60), the central hydrophobic region, also called non-amyloid component (NAC; amino acids: 61–95), and the acidic C-terminus (amino acids: 96–140) (Figure 1a). aSyn has attracted great attention in the neuroscience community because of its involvement in Parkinson’s disease (PD), the most common neurodegenerative movement disorder. The link of aSyn to PD is supported by genetic and neuropathological evidence: genetically, aSyn mutations, including point mutations, as well as aSyn gene (*SNCA*) duplication and triplication, cause familial forms of PD [3]; neuropathologically, accumulation of aggregated aSyn is an important protein component of inclusion bodies detected in neurons of PD patients. Occurrence of two distinct inclusion bodies, Lewy bodies (LBs) and Lewy neurites, which are localized in neuronal cell bodies and neurites, respectively, is a neuropathological hallmark of PD [4,5].

PD is clinically characterized by motor symptoms, including bradykinesia, rigidity, and resting tremor. The motor symptoms are primarily attributed to a selective and progressive loss of dopaminergic neurons in the substantia nigra pars compacta, located in the midbrain [6]. Extensive studies in recent decades have provided evidence attributing PD-related midbrain dopaminergic neurodegeneration to conformational alterations and aggregation of aSyn, probably due to the loss of its physiological function and/or the acquirement of aggregation-mediated toxic functions [7].

aSyn was initially identified in *Torpedo californica* and primarily detected in the nucleus and presynaptic nerve terminals [8]. Hence, the protein was named due to its presynaptic (“syn”) and nuclear localization (“nuclein”). Numerous studies have been conducted elucidating the physiological and pathological role of aSyn as a presynaptic protein and its interplay with dopamine homeostasis. aSyn has been reported to be involved in SNARE-complex assembly and synaptic vesicle recycling [9,10] and to interfere with dopamine synthesis, release, and reuptake [11]. In contrast to the intensively studied presynaptic functions of aSyn, the biology of nuclear aSyn is less investigated. In particular, the role of nuclear aSyn in neurodegeneration is not yet determined. The few studies available regarding the physiological function of nuclear aSyn demonstrated that aSyn in the nucleus binds to DNA as well as histones, and is involved in DNA repair and transcriptional regulation [12,13,14]. Several studies addressed the toxicity of nuclear aSyn, generating, however, inconsistent results [13,14,15,16,17,18]. The data published so far strongly suggest the existence of diverse species of aSyn with various assembly states within the nucleus [12]. Moreover, nuclear and cytosolic aSyn may also have different biochemical characteristics [13,14,16,17,18,19]. This complexity makes the study on nuclear aSyn difficult, partly because of the lack of antibodies, which sensitively and robustly recognize nuclear aSyn [19]. Furthermore, since aSyn is abundant in the cytoplasm, in particular enriched in presynaptic terminals, the question arises whether there is a cross-talk between aSyn in the nucleus and the cytoplasm, including neuronal processes, in health and disease. This essential question regarding the physiopathology of nuclear aSyn remains poorly understood.

In addition to preferential loss of midbrain dopamine neurons (mDANs) associated with PD, different neuronal compartments may have various susceptibilities to dysfunction. Accumulating evidence has indicated that axonal degeneration precedes the loss of neuronal cell bodies during neurodegeneration [20,21]. In line with this notion, our recent study using mDANs differentiated from human-induced pluripotent stem cells (hiPSCs) derived from PD patients carrying a heterozygous *SNCA* gene duplication (*SNCA^Dupl^*) demonstrated neuritic deficits, accompanied by an elevated level of aggregated aSyn [22]. Hence, we hypothesize that aggregated aSyn is enriched in the neurites, where it compromises neuritic homeostasis and consequently leads to neuritic dysfunction and neuronal loss.

In the present study, we focused on nuclear–somatic–neuritic shuttling of aSyn during neuronal differentiation by using an anti-aSyn antibody (Nu-aSyn-C) targeting the C-terminus of aSyn. This antibody exhibits an especially strong immunoaffinity to nuclear aSyn in proliferating cells and preferentially binds to assembled or aggregated aSyn. Employing two distinct human mDAN cell models, Lund human mesencephalic cells (LUHMES) and hiPSCs, we observed a characteristic redistribution of Nu-aSyn-C-positive aSyn species from the nucleus towards neurites in differentiating neurons. Importantly, this spatial redistribution was remarkably increased in differentiated mDANs carrying *SNCA^Dupl^*. Our study presents novel evidence about cell compartment-associated dynamics of distinct aSyn species during neuronal differentiation and provides a novel insight into altered aSyn subcellular distribution in PD-derived neurons.

## 2. Materials and Methods

### 2.1. Recombinant Synucleins and Preformed aSyn Oligomers

Recombinant human aSyn as well as bSyn and gSyn were generated as described in our previous study [23]. Human aSyn was expressed in *E. coli* BL21 (DE3) plysS competent cells (Novagen) transformed with a human aSyn PT7-7 plasmid vector (gift from Dr. Hilal Lashuel, Addgene plasmid #36046; RRID: Addgene_36046 [24]). Using the PT7-7 vector backbone, plasmid vectors carrying the coding region of human bSyn or gSyn gene were generated. Preformed aSyn oligomers were produced by incubating aSyn with 4-hydroxy-2-nonenal, as described in previous studies [23,25]. This type of aSyn oligomers has been shown to be partly SDS-stable, which can be detected by SDS-PAGE and Western blot (WB) [23].

### 2.2. H4 Neuroglioma Cells

Human neuroglioma cells (ATCC, HTB-148) were cultured in Opti-MEM™ Reduced Serum Medium, GlutaMAX™ Supplement (Thermo Fisher Scientific, Waltham, MA, USA) supplemented with 10% FCS (Sigma-Aldrich, St. Louis, MO, USA) and 1% Penicillin-Streptomycin (Thermo Fisher Scientific) at 37 °C in humidified 95% air and 5% CO_2_ atmosphere.

### 2.3. LUHMES Cells

Lund human mesencephalic (LUHMES) cells were a kind gift from M. Leist and D. Scholz, University of Konstanz, Germany. The cells were cultured at 37 °C in a humidified 95% air, 5% CO_2_ atmosphere. Cells were proliferating in advanced growth medium DMEM/F12 (Sigma-Aldrich) containing 1% N2 supplement (Life Technologies, Carlsbad, CA, USA), 40 ng/mL basic fibroblast growth factor (PeproTech, Hamburg, Germany), and 2 mM L-GlutaMAX (Life Technologies) on a culture flask (Nunclon DELTA surface, Thermo Fisher Scientific) coated with geltrex. The cells were washed once prior to detach them with trypsin and resuspended in DMEF/F12 to inactivate proteolytic activity of trypsin. For neuronal differentiation, the advanced growth medium was replaced by differentiation medium, containing DMEM/F12 with 1% N2 supplement, 2 mM L-GlutaMAX, 2 µg/mL tetracycline (Life Technologies), 1 mM dbcAMP, Sigma-Aldrich), and 20 ng/mL glial cell-derived neurotrophic factor (GDNF, PeproTech). Two days after pre-differentiation, cells were replated using TrypLE Express (Life Technologies). After 8 days of differentiation, neurons were maintained in neural basal medium with 1% N2 supplement, 1% B27 supplement (Life Technologies), 1 mM dbcAMP, and 20 ng/mL GDNF. For immunocytochemistry (ICC), pre-differentiated cells were seeded on coverslips in multi-well plates (Nunclon DELTA surface). Coverslips were edged with 37% HCl for 20 min at room temperature (RT), while gently rocking on a shaker and washed twice with water before coated with 500 µg/mL geltrex for at least 3 h in the incubator.

### 2.4. Differentiation of mDANs from hiPSCs

For generating hiPSC, human dermal fibroblasts derived from healthy individuals were reprogrammed using retroviral transduction of the transcription factors OCT3/4, c-MYC, SOX2, and KLF4 as previously described [26]. HiPSC clones (ID: UKERi1E4-R1-003, UKERi33Q-R1-006, UKERi33Q-R2-016, UKERi82A-S1-017 and UKERiO3H-R1-001) from four healthy individuals were obtained from the Stem Cell Core Unit of the Friedrich-Alexander-University Erlangen-Nürnberg (FAU). Written informed consents were, respectively, received from donors prior to inclusion in the study at the Movement Disorder Clinic at the Department of Molecular Neurology, University Hospital Erlangen (Erlangen, Germany). Experiments using hiPSC-derived cells were conducted in accordance with the Institutional Review Board Approval (Nr. 259_17B). Two hiPSC lines from a PD *SNCA^Dupl^* patient were provided by Dr Roybon (lines CSC-1A and CSC-1D, previously described in [27]). The generation of these hiPSC lines were approved by the Swedish work environment authority (registered under the number 20200–3211). The mDANs from CSC-1A were characterized in our previous study [28].

Differentiation of mDANs was performed based on a small molecule-induced hiPSC differentiation protocol [29]. Specifically, after detaching hiPSCs using collagenase IV (Thermo Fisher Scientific, Grand Island, New York, NK, USA), cell colonies were resuspended in human embryonic stem cell (hESC) medium (80% KO-DMEM, 20% KO serum replacement, 1% nonessential amino acids, 1% Penicillin-Streptavidin (all from Thermo Fisher Scientific), and 1 mM ß-Mercaptoethanol (Sigma-Aldrich), containing the small molecules 1 μM LDN-193189, 10 μM SB, 3 μM Chir, and 0.5 μM Purmorphoamine (PMA, all from Tocris Bioscience, Wiesbaden-Nordenstadt, Germany)) and seeded on ultra-low adhesion plates. After two days, medium was hanged to N2B27 medium (50% DMEM/F12, 50% Neurobasal Medium, 1:200 N2, 1:100 B27 (all from Thermo Fisher Scientific)) supplemented with the small molecules as given above. On day 4, the medium was replaced by small-molecule neural precursor cell (smNPC) medium (N2B27 medium supplemented with 3 µM Chir, 0.5 µM PMA and 150 µM ascorbic acid (AA, Sigma-Aldrich)). Subsequently, to generate smNPCs, cell colonies were replated after two more days on 12-well plates, coated with geltrex (Thermo Fisher Scientific), in smNPC medium supplemented with Rho kinase inhibitor Y27532 (Tocris Bioscience).

For mDANs differentiation, smNPCs (d0) were seeded in 12-well plates (Nunclon DELTA surface, Nunc, Roskilde, Denmark) coated with 500 µg/mL Matrigel (VWR International GmBH, Darmstadt, Germany). Two days after passaging, the medium was changed to N2/B27 medium with 100 ng/mL FGF8 (PeproTech), 1 µM PMA, and 200 µM AA. On day 8, maturation medium (N2/B27 with 100 ng/mL FGF8, 1 ng/mL GDNF, 10 ng/mL TGF-β3 (PeproTech), 200 µM AA, and 500 µM dibutyryl cyclic AMP (dbcAMP)) was used for differentiation of mDANs. One day after changing to maturation medium, the cultures were splitted at a 1:6 ratio and seeded in 6-well plates (Nunclon DELTA surface) coated with 500 µg/mL Matrigel. Afterwards, cells were maintained in maturation medium for up to 21 days.

### 2.5. Immunocytochemistry

Cells were fixed with 4% paraformaldehyde (PFA) in PBS for 15 min at RT, washed 3 times with PBS, and pre-treated with phosphate buffered saline (PBS) containing 1% BSA/0.5% Triton X-100. Incubation with primary antibodies was carried out overnight at 4 °C and with secondary antibodies for 1–2 h at RT. Cells on coverslips were mounted with Aqua-Poly/Mount (Polysciences Inc., Warrington, UK). Images were taken with a 20× (0.8, Apochromat), a 63× (1.40 oil, Plan-Apochromat) or a 100× (1.3 oil, Plan-Neofluar) objective as stacks of multiple optical sections at a Zeiss Axio Imager Z1 equipped with an ApoTome or with a Zeiss LSM 710 (Zeiss, Oberkochen, Germany). Projections were calculated with the Zen 2013 software (Zeiss) and 3D reconstructions were generated using Imaris (Bitplane, Zurich, Switzerland). For quantification of subcellular aSyn levels, signal intensities of aSyn stainings were determined with the Zen 2013 software for the nuclear and extra-nuclear regions (including the soma and neurites).

### 2.6. Electron Microscopy

For electron microscopy (EM), cells were cultured on lumox cell culture dishes (Sarstedt AG & Co., Nümbrecht, Germany) and fixed in 4% PFA, 2.5% glutaraldehyde, and 0.1 M cacodylate buffer (pH 7.4) for 15 min at 37 °C. After PBS wash, cells were fixed with 1% OsO4 in cacodylate buffer for 1 h at RT followed by a second wash with 0.1 M cacodylate buffer. Cells were dehydrated using an acetone series and mounted in Epon resin (Fluka, Buchs, Switzerland). Blocks with embedded cells were sectioned with a vibratome VT 1000 S (Leica, Wetzlar, Germany) and ultrathin sections were stained with uranyl acetate and lead citrate. The sections were examined and photographed with a Zeiss EM10 electron microscope (Zeiss) and a Gatan SC1000 OriusTM CCD camera (GATAN, Munich, Germany) in combination with the DigitalMicrograph™ 3.1 software (GATAN, Pleasanton, CA, USA).

### 2.7. Mouse Brain Tissue

Breeding of C57BL6N (WT) mice and *SNCA* knockout mice (KO) mice [30] were described in detail in [31]. For biochemical analysis, whole brain tissue was dissected from the mice and snap frozen immediately after the dissection. For immunohistochemistry (IHC), brain tissue was fixed in 4% PFA overnight and next transferred into 30% sucrose. Brain hemispheres were coronally sectioned (40 µm) and kept in a cryoprotection solution (21.3% glycerol, 25% ethylene glycol in 0.1 M phosphate buffer) at −20 °C until staining. Animals were kept under a standard 12:12 light:dark cycle and had free access to food and water ad libitum. All procedures with animals were approved by the local Animal Welfare and Ethics committee of Bavaria, Germany (TS-09/11 and TS 3/2020).

### 2.8. Immunohistochemistry

PFA-fixed coronal tissue sections were rinsed 3 times for 5 min each with Tris buffered saline (TBS) with 0.05% TritonX-100 (TBS/0.05% TX-100) and subsequently incubated in citrate buffer (0.1 M citric acid and 0.1 M sodium citrate in H_2_O) for 30 min at 80 °C for antigen retrieval. Afterwards, tissue sections were cooled down to RT. After washing with TBS/0.05% TX-100 (3 × 5 min), the sections were incubated in blocking solution (3% donkey serum (Pan Biotech), 0.3% TBS/TX-100) for 2 h at RT to reduce unspecific antibody binding. Next, immunostaining of the brain tissue was performed overnight at 4 °C on a shaker (75 rpm) using a primary antibody diluted in blocking solution overnight. On the next day, tissue sections were washed with TBS/0.05% TX-100 (6 × 10 min) and subsequently incubated with a secondary antibody diluted in blocking solution for 1 h at RT. Thereafter, tissue sections were rinsed in TBS/0.05% TX-100 (3 × 5 min), counterstained using 4′,6-diamidino-2-phenylindole (DAPI, Sigma-Aldrich, dilution 1:10,000 in TBS) for 10 min at RT and rinsed another 3 times in TBS/0.05% TX-100. Finally, tissue sections were mounted on glass slides using ProlongTM Gold antifade reagent (Life Technologies).

### 2.9. Preparation of Cell and Tissue Lysates

Cells were homogenized in TBS with 1% TX-100 containing protease inhibitor cocktail (cOmplete, EDTA free) and PhosSTOP™ (both from Roche, concentrations according to manufacturer’s recommendation) using a B. Braun Potter S Homogenizer (Sartorius AG, Göttingen, Germany). To further lyse the cells, the cell homogenates were mixed with 4×RIPA buffer (200 mM Tris/HCl pH 8.0, 450 mM NaCl, 5 mM EDTA, 4% NP40, 2% sodium deoxycholate, 0.4% SDS) in a ratio of 3:1 for 30 min on ice. Subsequently, samples were centrifuged at 10,000× *g* for 10 min at 4 °C. Protein content of the supernatant was determined using bicinchoninic acid (BCA) assay (Thermo Fisher Scientific).

Mouse brain tissue was homogenized in 1 × RIPA buffer (50 mM Tris/HCl pH 8.0, 150 mM NaCl, 5 mM EDTA, 1% NP40, 0.5% sodium deoxycholate, 0.1% SDS) containing protease inhibitor cocktail. The brain lysate was next centrifuged at 20,000× *g* for 10 min at 4 °C. The supernatant was collected and protein concentration was determined using BCA assay.

### 2.10. Subcellular Fractionation

To separate nuclear and cytosolic proteins, 9 × 10^6^ cells were homogenized in 150 µL fractionation buffer (20 mM HEPES, pH 7.4, 250 mM Sucrose, 1 mM EDTA, 1 mM EGTA, 1 mM DTT in the presence of protease inhibitor cocktail) by passing the cells through a 27 gauge needle 10 times on ice. The total cell homogenate was centrifuged at 1000× *g* for 10 min at 4 °C. The first pellet (P1) and supernatant (S1) were separated, and P1 was next resuspended in 150 µL fractionation buffer, passed through a 27 gauge needle again, and centrifuged at 1000× *g* for 10 min at 4 °C. The supernatant (S2) was discarded, and the pellet (P2) contained nuclear proteins further dissolved in 150 µL TBS buffer with 0.1% SDS. The S1 fraction was centrifuged at 100,000× *g* for 1 h at 4 °C by using an ultracentrifuge (Sorvall wX+ULTRA SERIES Centrifuge, Thermo Scientific). The resulting supernatant (S2′) contained cytosolic proteins. For detection of subcellular distribution of proteins, equal volume of cytosolic and nuclear fractions was utilized for WB or dot blot analysis.

### 2.11. SDS-PAGE, Western Blot and Dot Blot

For WB, 20 μg total protein from cells or brain tissue, or 1 µg recombinant aSyn or preformed oligomers were mixed with LDS sample buffer and reducing agent (Thermo Fisher Scientific) in a dilution based on manufacturer’s recommendations. Afterwards, the samples were heated at 70 °C for 10 min. For SDS-PAGE, samples were loaded on precast gels NuPage 4–12% Bis-Tris or Bolt 4–12% Bis-Tris Plus (Thermo Fisher Scientific). For protein transfer, the gel was blotted on an Immobilon^®^-FL PVDF membrane (Merk Millipore, Darmstadt, Germany). After blotting, the PVDF membrane was incubated with 4% PFA for 15 min, then thoroughly rinsed with TBS-T buffer (TBS with 0.1% Tween 20). For loading control of total protein, the gel was stained with Coomassie Staining solution (10% ethanol, 5% aluminiumsulphate, 2% phosphoric acid, 0.02% Coomassie Brilliant blue-G250 (CBB, Sigma-Aldrich)) for 12 h.

For dot blot, a nitrocellulose membrane (GE Healthcare, Life science, Amersham Protran, 0.2 µm) was placed into a dot blot chamber (Minifold Dot-Blot System, Schleicher & Schuell, Dassel, Germany). Amounts of 10–20 µg total protein from brain tissue, 12.5–200 ng of recombinant synucleins, or 5 µL cytosolic or nuclear fraction (refer to “subcellular fractionation” for preparation) were loaded on the membrane. After drying for 2 h at RT, the membrane was treated with 4% PFA for 15 min at RT, then rinsed with TBS-T 3 times for 5 min, followed by blocking with 1% BSA in TBS-T for 1 h at RT. Loading of the total protein was controlled by using Direct Blue 71 (Sigma-Aldrich) according to Hong et al. [32].

For immunostaining, the WB or dot blot membrane was blocked with 1% BSA in TBS-T for 1 h at RT. Followed by incubation with a primary antibody for 1 h at RT, the membrane was rinsed with TBS-T 3 times for 5 min. Afterwards, the membrane was incubated with a fluorescence-conjugated secondary antibody for 1 h at RT. After removing the secondary antibody by washing with TBS-T (3 × 5 min), the membrane was dried in the dark at RT. Signal detection and imaging were performed by using Fusion Fx7 (PEQLAB). Signal intensities were measured by using Image Lab Software (Version 6.0.1, BioRad, Hercules, CA, USA) or Bio1D (PEQLAB).

### 2.12. Antibodies

The following antibodies were used for immunohistochemistry (IHC), ICC, WB and dot blot:

Primary antibodies. Monoclonal rat anti-dopamine transporter (DAT, #MAB369, Millipore, Temecula, CA, USA, ICC 1:5000); monoclonal mouse anti-GAPDH G9 (#sc-365062, Santa Cruz Biotechnology, dot blot 1:2000); monoclonal rabbit anti Histone H3 D1H2 (#4499, Cell Signaling Technology, Danvers, MA, USA, dot blot 1:2000); polyclonal guinea pig anti-NeuN (#266004, Synaptic Systems, ICC 1:500), polyclonal rabbit anti-aSyn Nu-aSyn-C (#AX100078 OriGene Technologies, Rockville, MD, USA, IHC 1:100, ICC 1:150, WB/dot blot 1:500); monoclonal mouse anti-aSyn Syn1 (#610787, BD Biosciences, San Jose, CA, USA, ICC 1:100, WB/dot blot 1:1000); monoclonal mouse anti-aSyn LB 509 (#ab27766, Abcam, Cambridge, UK, dot blot 1:1000); monoclonal rabbit anti-aSyn phosphorylated on serine 129, EP1536Y (#ab51253, Abcam, dot blot, 1:1000); polyclonal guinea pig anti-Tau (#314004, Synaptic Systems, ICC 1:100); monoclonal mouse anti-β-III-tubulin 2G10 (#78078, Abcam, ICC 1:200, WB 1:1000); and polyclonal rabbit anti-tyrosine hydroxylase (TH, #AB152, Millipore, Billerica, MA, USA, ICC 1:1000).

Secondary antibodies. Alexa Fluor 488 goat anti-rabbit, anti-mouse, and anti-guinea pig IgG conjugates (Molecular Probes, Eugene, OR, USA, ICC 1:500); Fluor 568 goat anti-rabbit and anti-mouse IgG conjugates (Molecular Probes, ICC 1:500); Cy3 goat anti-mouse and anti-rabbit IgG conjugates (Dianova, Hamburg, Germany, 1:200–1:1000), Cy5 goat anti-mouse and anti-rabbit IgG conjugates (Dianova, 1:100–1:1000), and HRP goat anti-rabbit and anti-mouse IgG conjugates (Sigma-Aldrich or Dianova, 1:10,000). For ICC, nuclei were stained with DAPI (1:50,000).

### 2.13. Statistics

GraphPad Prism version 5.03 (GraphPad Software, Inc., San Diego, CA, USA) was used for statistical analyses. Statistical tests for determining significant differences are given in the legends. *p*-values < 0.05 were considered statistically significant. All graphs are presented as the mean of independent experiments ± standard deviation (SD).

## 3. Results

### 3.1. The Nu-aSyn-C Antibody Exhibits a High Immunoaffinity to Nuclear aSyn and Preferentially Binds to Aggregated aSyn

The rabbit polyclonal anti-aSyn Nu-aSyn-C antibody was generated using a synthetic peptide near the C-terminus of human aSyn as an antigen (Origen, catalog number TA354105, former Acris catalog number AP15807, Figure 1a). To assess antibody specificity, we compared the immunocytochemical staining pattern of this antibody in brain tissue from wild-type (WT) and aSyn knockout (KO) mice with that of anti-aSyn Syn1 antibody. Syn1 is generated using a rat aSyn fragment (amino acids: 15–123) and is frequently used to characterize aSyn for biochemical analyses and ICC or IHC [33,34]. Like Syn1, the Nu-aSyn-C antibody only showed specific immunocytochemical signals in WT, but not in KO brains, utilizing either denaturing SDS-PAGE-based WB or native dot blot analysis (Figure 1b,c). Concordantly, immunocytochemical staining with the Nu-aSyn-C antibody revealed specific and defined structures in brain tissue sections from WT mice (Figure 1d and Figure S1). In KO brains, only unspecific background signals were detected. As a-, b-, and gSyn share high sequence homology in particular in their N-terminal regions, we also tested whether the Nu-aSyn-C antibody recognizes additional members of the synuclein family other than aSyn. Dot blot analysis demonstrated that the Nu-aSyn-C antibody does not cross-react with b- and gSyn. In conclusion, the Nu-aSyn-C antibody shows a high specificity to aSyn.

**Figure 2:**
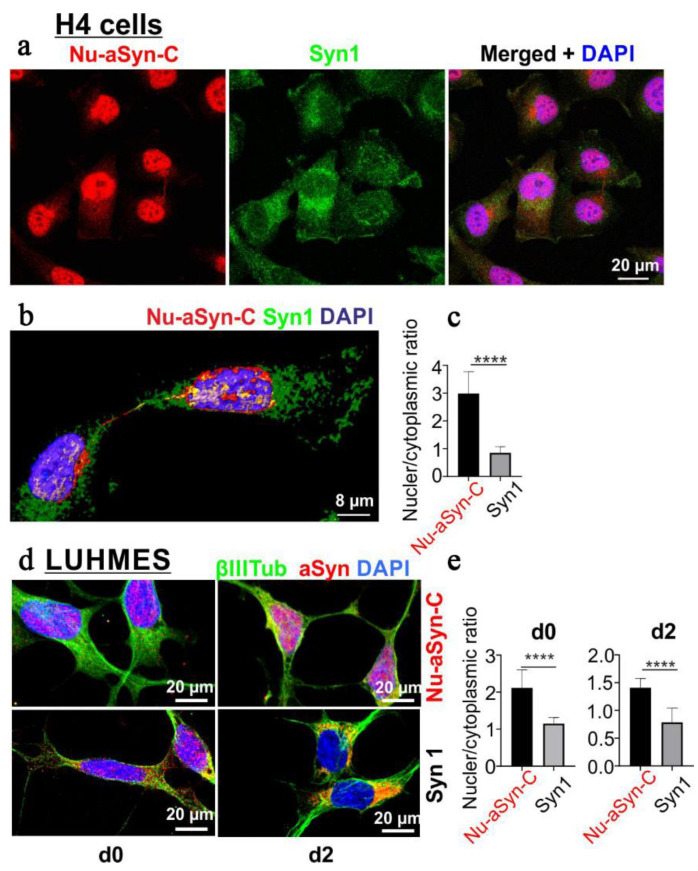
Labelling of aSyn with Nu-aSyn-C and Syn1 antibodies in proliferating H4 and LUHMES cells (d0), as well as in LUHMES cells after 2 days of differentiation. Nu-aSyn-C immunosignals are predominantly present in the nucleus, whereas Syn1 immunosignals are detected to a larger extent in the cytosol. (**a**) ICC analysis of aSyn distribution in H4 cells detected by Nu-aSyn-C and Syn1 antibodies. (**b**) A 3D reconstruction of representative proliferating H4 cells. (**c**) Ratio of nuclear versus cytoplasmic immunosignal intensity detected by Nu-aSyn-C and Syn1 antibodies, respectively. Data represent the mean ± SD from 35 cells for each antibody. Statistics: Mann–Whitney test; **** *p* < 0.001. (**d**) aSyn distribution patterns recognized by Nu-aSyn-C and Syn1 antibodies, respectively, in proliferating (d0) and differentiating LUHMES cells (d2). (**e**) Quantification of the ratios of nuclear and cytoplasmic immunosignal intensity. Data represent the mean ± SD from 11–21 cells for each antibody. Statistics: Mann–Whitney test; **** *p* < 0.001.

We next characterized the binding patterns of Nu-aSyn-C and Syn1 antibodies in cells. We performed ICC using immortalized H4 neuroglioma cells as well as LUHMES cells at the proliferating stage (d0) and after 2 days of differentiation (d2). Remarkably, the immunosignals of Nu-aSyn-C and Syn1 antibodies showed distinct distribution patterns (Figure 2): In proliferating H4 and LUHMES cells (d0) as well as in LUHMES cells differentiated for 2 days (d2), the Nu-aSyn-C antibody predominantly recognized nuclear aSyn, while the Syn1 antibody detected, to a larger extent, cytosolic aSyn.

**Figure 3:**
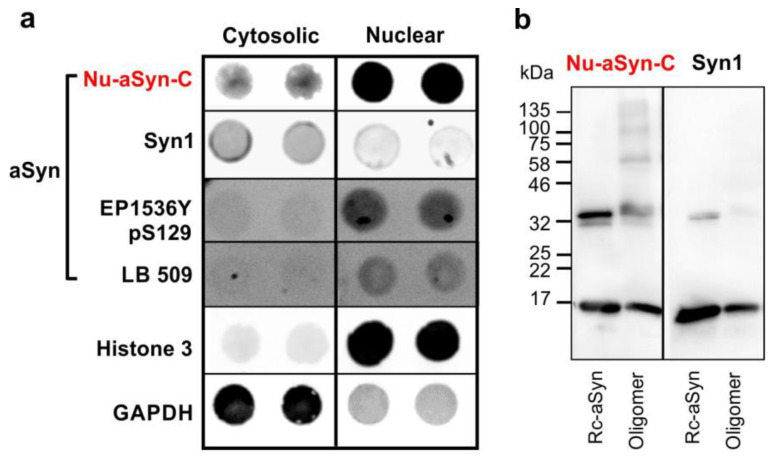
(**a**) Dot blot analysis of enriched cytosolic and nuclear fractions from H4 cells using different anti-aSyn antibodies, including Nu-aSyn-C, Syn1, EP1536Y (specific for phosphorylated aSyn on serine 129), and LB 509 (recognizing aggregated aSyn). GAPDH and histone 3 serve as protein markers of the cytosol and the nucleus, respectively. In H4 cells, nuclear aSyn exhibits a higher phosphorylated and aggregated status than cytosolic aSyn. (**b**) WB analysis of human recombinant aSyn (Rc-aSyn) and preformed aSyn oligomers. The Nu-aSyn-C antibody preferentially recognizes higher-molecular-weight oligomers.

To verify the distinct distribution patterns detected by ICC with Nu-aSyn-C and Syn1 antibodies, we enriched nuclear and cytoplasmic fractions from H4 cells by biochemical fractionations followed by native dot blot and denaturing SDS-PAGE/WB analyses. In line with the ICC results, dot blot and WB analyses both confirmed a higher affinity of the Nu-aSyn-C antibody to nuclear aSyn, as compared to cytosolic aSyn (dot blot, Figure 3a; WB, Figure S2a). Interestingly, WB analysis using the Nu-aSyn-C antibody detected not only aSyn monomers but also abundant high-molecular-weight immunopositive species in the whole-cell homogenate and in the nuclear fraction. This was remarkably different to the WB pattern detected by using Syn 1, which recognized the aSyn monomer as the most prominent band on WB (Figure S2b). These results suggest the existence of different species of aSyn in the nucleus and cytosol. To confirm this consumption, we probed the cytosolic and nuclear extracts from H4 cells with the EP1536Y antibody, which is directed against phosphorylated aSyn on serine 129 (EP1536Y, Figure 1a) and the LB 509 antibody, which was generated using enriched LBs as antigens and recognizes human aSyn residues 115–122 [4,35]. LB 509 was shown to strongly bind to amyloid fibrils and aggregated aSyn filaments extracted from postmortem brain tissue of patients with multiple system atrophy [35,36]. Since denaturing SDS-PAGE may change aSyn conformation, we used the non-denaturing dot blot approach. We observed a higher level of phosphorylated aSyn and LB 509 immunosignal intensity in the nuclear fraction compared to the cytosolic fraction (Figure 3a).

To further verify that the Nu-aSyn-C antibody that strongly labels nuclear aSyn has a high binding affinity to assembled or aggregated aSyn, we tested the binding of Nu-aSyn-C and Syn1 antibodies to human recombinant aSyn and preformed, SDS-stable aSyn oligomers (Figure 3b). Both antibodies recognized monomeric (~17 kDa) and dimeric (~32 kDa) aSyn on WB, but like the WB patterns in H4 cells (Figure S2), the Nu-aSyn-C antibody detected high-molecular-weight oligomeric aSyn species far better than the Syn1 antibody. In summary, biochemical cellular fractionation combined with the application of different anti-aSyn antibodies revealed cell compartment-associated aSyn species with distinct biochemical characteristics. Our findings suggest an enrichment of phosphorylated and aggregated aSyn in the nucleus of proliferating H4 cells. Consistently, the Nu-aSyn-C antibody, which exhibits a high affinity to nuclear aSyn, also has a strong binding capacity to aggregated aSyn.

### 3.2. Nu-aSyn-C-Positive aSyn Species Are Redistributed from the Nucleus to the Soma, and Further to Neurites during Neuronal Differentiation

**Figure 4:**
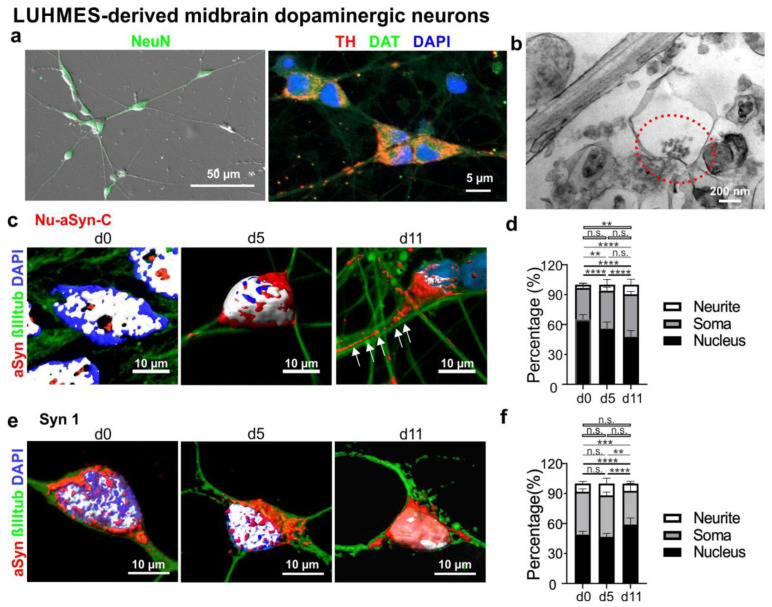
Characterization of mDANs differentiated from LUHMES cells. (**a**) Representative neurons differentiated for 5 days show the expression of the neuronal marker NeuN (left, green) and dopaminergic neuronal markers TH (red) and DAT (green). (**b**) Representative EM image with a synaptic site (red circle) in LUHMES cells differentiated for 35 days. (**c**,**e**) Representative 3D reconstructed fluorescence images of LUHMES cells at the proliferating stage (d0), and differentiated for 5 (d5) and 11 days (d11) labelled with either the Nu-aSyn-C (**c**) or Syn1 antibody (**e**), βIIItub and DAPI. (**d**,**f**) Quantification of aSyn distribution (in %) in the nucleus, soma, and neurites, labeled with either the Nu-aSyn-C (**d**) or Syn1 (**f**) antibody. In the 3D reconstructed images (**c**,**e**), nuclear localization of aSyn is depicted in white due to the special morphology of LUHMES cell bodies characterized by a close arrangement of the nucleus and the surrounding soma. The Nu-aSyn-C antibody detects a strong localization of aSyn in the nucleus of proliferating cells (d0). Upon differentiation (d5–d11), aSyn signal intensity in the soma and neurites significantly increases. This shift of the aSyn signal revealed by the Nu-aSyn-C antibody is much less visible using the Syn1 antibody. For quantification (**d**,**f**), data represent the mean ± SD from 9–19 cells of each time point measured in three independent experiments. Statistics: Two-way ANOVA, Tukey’s multiple comparisons test, n.s.: not significant, ** *p* < 0.005, *** *p* < 0.0005, and **** *p* < 0.0001. Black lines: comparisons of nuclear signal intensity; grey lines: comparisons of soma signal intensity; white lines: comparisons of neurite signal intensity.

Since the Nu-aSyn-C antibody preferentially recognizes aggregated aSyn as well as nuclear aSyn in proliferating cells, we next asked whether Nu-aSyn-C-positive species are associated with distinct subcellular compartments during neuronal differentiation. To address this issue, we used at first LUHMES cells as a mDAN model. LUHMES cells are conditionally immortalized human fetal midbrain neurons that have the potential to differentiate into postmitotic neurons [23,37]. LUHMES cells differentiated for 5–11 days (Figure S3) expressed the neuronal marker NeuN as well as markers characteristic for dopaminergic phenotypes, such as TH and DAT (Figure 4a). EM analysis of LUHMES cells differentiated for up to 35 days showed the presence of putative synaptic sites, characterized by synaptic vesicle-like structures (Figure 4b). To determine subcellular localization of aSyn, we labelled proliferating LUHMES cells (d0) and cells differentiated for 5 (d5) and 11 days (d11) for aSyn (Nu-aSyn-C and Syn1 antibodies), neuronal marker class III beta-tubulin (βIIItub), and DAPI. Furthermore, 3D reconstructions were performed to distinguish aSyn in the nucleus from that in the soma or neurites (Figure 4c,e). After 5 days of differentiation, Nu-aSyn-C-positive immunosignals, which were predominantly present within the nucleus on d0, decreased in the nucleus and increased in the soma (Figure 4c,d). On d11, Nu-aSyn-C-positive signals along the neurites were significantly enhanced. Intriguingly, this Nu-aSyn-C-associated, differentiation-dependent shift of aSyn immunosignals from the nucleus to neurites was not detected in neurons labeled with Syn1 (Figure 4e,f), indicating a specific redistribution pattern related to Nu-aSyn-C-positive aSyn species.

**Figure 5:**
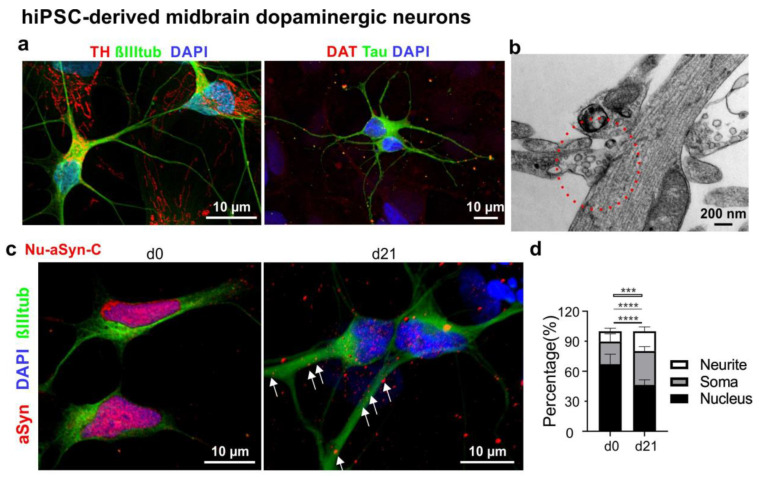
Characterization of mDANs differentiated from hiPSCs. (**a**) Representative neurons differentiated for 21 days show the presence of the neuronal markers βIIItub and Tau (green) as well as the dopaminergic neuronal markers TH and DAT (red). (**b**) Representative EM image of a putative synaptic site (red circle) with synaptic vesicles in hiPSC-derived neurons differentiated for 21 days. (**c**) Representative immunofluorescence images of proliferating smNPCs (d0) and neurons differentiated for 21 days (d21) labelled with the Nu-aSyn-C antibody, an anti-βIIItub antibody, and DAPI. Immunosignals for aSyn and DAPI were reconstructed in 3D to demonstrate the distribution pattern of aSyn. (**d**) Quantification of aSyn distribution (in %) in the nucleus, soma and neurites. The Nu-aSyn-C antibody detects aSyn in the nucleus of proliferating cells (d0). Upon differentiation (d21), aSyn signal intensity significantly decreases in the nucleus and increases in the soma and neurites. For quantification, data represent the mean ± SD of >16 cells of each time point measured in three independent experiments. Two hiPSC lines (UKERi33Q-R1-006 and UKERi1E4-R1-003) from two healthy individuals were used. Statistics: Two-way ANOVA, Tukey’s multiple comparisons test, *** *p* < 0.0005, and **** *p* < 0.0001. Black lines: comparisons of nuclear signal intensity; grey lines: comparisons of soma signal intensity; white lines: comparisons of neuritic intensity.

To verify the redistribution of Nu-aSyn-C-positive aSyn species during neuronal differentiation, we next performed ICC analysis of mDANs differentiated from hiPSCs using a small molecule-based differentiation protocol [22,29]. Neurons were differentiated from hiPSCs via small molecule-induced neural precursor cells (smNPCs) (Figure S3). Neurons differentiated from smNPCs for 21 days displayed neuronal markers, such as βIIItub and Tau, as well as specific makers for dopaminergic neurons, including TH and DAT (Figure 5a). Ultrastructural EM analysis of differentiated neurons showed distinct synaptic sites, comprising a presynaptic terminal with synaptic vesicles and a postsynaptic density (Figure 5b). Consistently with our observation in LUHMES cells, Nu-aSyn-C-positive species were predominantly present within the nucleus. In neurons differentiated for 21 days (d21), aSyn intensity in the nucleus was significantly attenuated, but within the soma and along the neurites it increased significantly (Figure 5c,d).

Interestingly, the redistribution of Nu-aSyn-C-positive aSyn species during dopaminergic neuronal differentiation, either in LUHMES- or hiPSC-based model, was also detected in primary rat hippocampal neurons (Figure S4).

### 3.3. Distribution of Nu-aSyn-C-Positive aSyn Species Is Shifted towards Neurites in SNCA^Dupl^ mDANs

**Figure 6:**
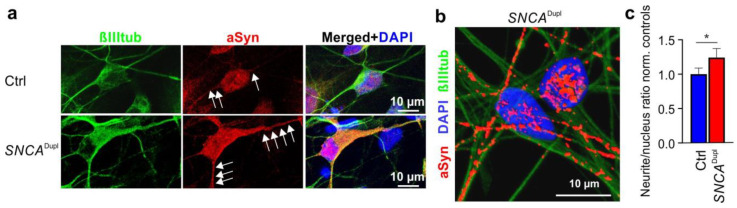
ICC analysis of aSyn distribution in mDANs from healthy controls (Ctrl) and a *SNCA^Dupl^* patient. (**a**) Representative immunofluorescence images of mDANs differentiated for 21 days labelled with the Nu-aSyn-C antibody, anti-βIIItub, and DAPI. The corresponding TH staining is shown in Figure S5. (**b**) Representative reconstructed 3D image highlights intense aSyn immunosignals along the neurites of *SNCA^Dupl^* mDANs. (**c**) Quantification of neurite/nucleus ratios of aSyn immunosignal intensity in *SNCA^Dupl^* mDANs normalized to the average ratio in control-derived neurons. For quantification, data represent the mean ± SD of >10 neurons of each hiPSC line in three independent experiments. Three hiPSC cell lines from three healthy individuals (UKERi33Q-R1-006, UKERiO3H-R1-001, and UKERi82A-S1-017) and two hiPSC lines (CSC-1A and CSC-1D) from the *SNCA^Dupl^* patient were used. Statistics: Two-way ANOVA, Tukey’s multiple comparisons test * *p* < 0.05.

Our previous studies on *SNCA^Dupl^* mDANs derived from PD patients demonstrated neuritic deficits [22]. Since the Nu-aSyn-C antibody preferentially binds to aggregated aSyn, we next asked whether the nuclear/neuritic distribution pattern in differentiated mDANs is altered in *SNCA^Dupl^* mDANs, underlying the observed neuritic phenotype. We analyzed Nu-aSyn-C immunosignal patterns in mDANs differentiated from hiPSC lines of healthy controls and a *SNCA^Dupl^* patient using ICC. An enhanced aSyn level in *SNCA^Dupl^* neurons has been previously shown in our extensive studies on different *SNCA^Dupl^* cell lines, including cells from the patient analyzed in this study [22,28,38]. As expected, *SNCA^Dupl^* neurons are characterized by a remarkably intense staining of aSyn due to high aSyn expression levels, when compared to neurons from healthy controls (Figure 6a). Conspicuously, Nu-aSyn-C immunosignals along the neurites were stronger in *SNCA^Dupl^* mDANs than in controls (Figure 6a, white arrows). This effect is especially evident in 3D constructed images of *SNCA^Dupl^* mDANs (Figure 6b). We next asked whether the strong neuritic Nu-aSyn-C immunosignals were due to a general increase in aSyn levels in these cells or a result of disproportionate increase. For this, we calculated the Nu-aSyn-C fluorescence intensity ratios in the neurites versus nucleus of *SNCA^Dupl^* mDANs and normalized the ratios against those of control cells. To ensure that dopaminergic neurons were analyzed, we only focused on the neurons that were positive for both βIIItub and TH (Figure 6a and Figure S5). We detected a significantly increased neuritic/nuclear ratio of aSyn in *SNCA^Dupl^* mDANs (Figure 6c). This finding indicates a disproportional nuclear to neuritic shift of possibly aggregated aSyn, most likely triggered by a cell autonomous overexpression of aSyn.

## 4. Discussion

Since the discovery of aSyn in presynaptic nerve terminals and in the nucleus [8], the relationship of nuclear and neuritic aSyn as well as the assembly states of aSyn in distinct subcellular compartments have remained elusive. Using the specific Nu-aSyn-C antibody, we demonstrated here a dynamic redistribution of Nu-aSyn-C immunosignals from the nucleus to the soma at earlier, and from the soma to neuritic compartments at later differentiation stages. Interestingly, this redistribution pattern of aSyn appears to be common for developing neurons regardless of the neurotransmitter phenotype. We observed this effect not only in mDANs differentiated from human LUHMES cells or hiPSCs, but also in primary rat hippocampal cell culture comprised of glutamatergic and GABAergic neurons. It should be especially noted that the nuclear–neuritic shift of aSyn is only limited to Nu-aSyn-C immunopositive aSyn species in our study. ICC labeling with the Syn1 antibody could not recapitulate the characteristic redistribution of aSyn detected by the Nu-aSyn-C antibody in identical LUHMES mDANs. In summary, our findings indicate that distinct aSyn species, which are preferentially recognized by the Nu-aSyn-C antibody, translocate from the nucleus to the neurites via the soma in developing neurons.

Existence of various aSyn species with different conformations and assembly states, in both physiological and pathological contexts, has been supported by findings of extensive studies on aSyn structure and functional properties. aSyn was initially believed to be a physiologically unfolded, thermostable monomer, which is able to adopt an alpha helical structure upon binding to lipid membranes [39,40,41]. Studies of recent years also indicated that aSyn may physiologically occur in alpha helical multimeric forms, which are suggested to be protective against pathological aggregation of aSyn and promote SNARE assembly [42,43,44]. Moreover, numerous neurotoxic aSyn aggregated forms have been reported in vitro, in cells and in vivo. These include aSyn oligomers, protofibrils, and fibrils, which are characterized by increased beta sheet structures and decreased solubility [45,46]. Pathological aSyn species have been shown to differ in the type of assemblies they form, structural characteristics, and propagation propensity (i.e., the ability to induce conformational changes and aggregation of physiological aSyn forms) [47,48]. In this study, spatial and temporal analysis using antibodies recognizing various aSyn epitopes provide novel evidence for the presence of particular Nu-aSyn-C-positive aSyn species in the nucleus of proliferating cells and their nuclear to neuritic translocation during neuronal differentiation. Interestingly, comparisons of the Nu-aSyn-C antibody and the Syn1 antibody demonstrated distinct WB patterns either in vitro using recombinant aSyn species or in the whole-cell homogenate of H4 cells. Results of both approaches point towards a stronger binding capacity of the Nu-aSyn-C antibody to high-molecular-weight species as compared to the Syn1 antibody, suggesting that Nu-aSyn-C-positive aSyn species have a high assembly state. It is noteworthy to mention that the Nu-aSyn-C-positive, high-molecular-weight species detected in H4 cells using denaturing SDS-PAGE/WB analysis should be interpreted with caution. The current study cannot exclude the possibility that the high-molecular-weight species are in part heterooligomers of aSyn and other proteins in the cell.

To gain further insight into compartment-specificity of aSyn, we performed biochemical cell fractionation of H4 cells to separate nuclear and cytosolic proteins followed by native dot blot analysis using a panel of aSyn antibodies. The result revealed a clear difference between nuclear and cytosolic aSyn. In concordance with the results from our ICC analysis, nuclear aSyn showed a remarkably higher affinity to the Nu-aSyn-C antibody and a lower binding to the Syn1 antibody, while cytosolic aSyn exhibits opposite binding affinity to these antibodies. Moreover, we demonstrated that nuclear aSyn has a higher phosphorylation and aggregation level than cytosolic aSyn. These findings are in agreement with a previous study of Pinho et al., 2019 [12], demonstrating the presence of phosphorylated and aggregated aSyn in the nucleus. Using different cell models and postmortem brain samples, this study showed that phosphorylation of aSyn modulates its nuclear localization and its role in the nucleus as a regulator of gene transcription. It should be noted that different conformations of nuclear and cytosolic aSyn indicated by our epitope dot blot analysis could also be a result of interaction of aSyn in distinct conformations with other proteins in the nucleus and cytosol, leading to exposure of different epitopes.

In total, the species characterization demonstrated in our study supports the existence of diverse aSyn species with different subcellular distribution and nuclear–neuritic shuttling patterns. Despite evidence of a high phosphorylation and assembly status, a definite conclusion about the precise structure of nuclear aSyn and its interactome cannot be drawn yet based on our current data. Posttranslational modifications (PTMs) are important modifiers of aSyn structure and function. Despite its small size, aSyn is modified by a large number of PTMs [7]. In the future, approaches to more precisely define PTMs (such as chemical modifications and truncation) of purified nuclear aSyn in combination with structural analysis of the folding and assembly states, as well as interaction partners, might help to better understand the role of nuclear aSyn and its regulatory pathways.

An interesting finding of this study relates to the high Nu-aSyn-C immunosignals in the nucleus of H4 cells. Naïve H4 neuroglioma cells are well known for their low endogenous aSyn expression levels, and are frequently used to study aSyn biology by overexpressing aSyn variants in these cells [12,34]. It is unlikely that the strong nuclear Nu-aSyn-C-positive immunosignals are due to non-specific staining of the Nu-aSyn-C antibody. Antibody specificity tests using recombinant synucleins, animal brain tissue from WT and aSyn KO mice, and complementary analytic approaches (immunochemistry of PFA-fixed samples, denaturing SDS-PAGE/WB and non-denaturing dot blot) confirmed a high specificity of this antibody to aSyn. Moreover, the strong labeling of nuclear aSyn was also confirmed in LUHMES cells at proliferating and early differentiating stages. Thus, the intense nuclear aSyn signals revealed by the Nu-aSyn-C antibody is probably a result of an especially high affinity of this antibody to nuclear aSyn, although aSyn levels are generally low in these cells. This result underlines the importance to use suitable antibodies to characterize aSyn in the nucleus. At the time we prepared this manuscript, the manufacture unfortunately halted the supply of the Nu-aSyn-C antibody. Nevertheless, our study highlights the relevance of C-terminal epitopes of aSyn as binding sites for the detection of nuclear aSyn. Consistently, the anti-phosphorylated aSyn antibody EP1536Y and the LB 509 antibody, which also bind nuclear aSyn in H4 cells, although to a lesser extent, recognize C-terminal amino acid residues (EP1536Y, phosphorylated serine 129; LB 509, epitope 115–122) as well. Along this line, several previous studies also implied the relevance of the C-terminus for the nuclear localization and function of aSyn. For example, amino acid residues 103–140 were shown to be indispensable for importin-mediated nuclear import [16]. Residues 96–140 were reported as the essential region for nuclear aSyn function in gene expression regulation [17]. An anti-aSyn antibody targeting amino acid residues 115–121 demonstrated an extensive staining of nuclear aSyn in normal rat brain neurons [19]. Hence, targeting C-terminal aSyn sequences should be taken into account in selection, screening or generation of antibodies in future studies on the role of nuclear aSyn.

In the present study, a redistribution of Nu-aSyn-C immunosignals from the nucleus towards neurites during neuronal differentiation was detected in different neuronal cell models. We speculate that this nuclear–neuritic translocation of distinct aSyn species is required for regular neuronal differentiation. In *SNCA^Dupl^* mDANs, aSyn overload significantly promotes the translocation of Nu-aSyn-C-positive species from the nucleus to neurites. The increased neuritic Nu-aSyn-C immunosignals are disproportionate and not simply due to aSyn overexpression. This disturbed nuclear–neuritic translocation of aSyn resembles the mislocalization of TAR DNA-binding protein 43 (TDP-43) from the nucleus to cytoplasm described in frontotemporal dementia (FTD) and amyotrophic lateral sclerosis [49,50]. Thus, our novel finding on aSyn distribution indicates a potential role of nuclear–neuritic shuttling in neurodegeneration, in particular in neuritic degeneration. Mounting evidence suggests that neuritic degeneration and dysfunction temporally precedes the loss of neuronal cell bodies in different neurodegenerative disorders [20,51]. In our previous studies on the characterization of hiPSC-derived neurons from *SNCA^Dupl^* PD patients, we observed in these neurons an increased level of total and aggregated aSyn [22,28,38], accompanied by neuritic deficits, such as impairments in neurite outgrowth [22] and a reduced axonal transport activity [38]. Since dynamic microtubule organization is essential in neurite development, the interplay between aggregated aSyn and microtubule network could be a possible mechanism that modulates neurite integrity in PD. Indeed, a direct interaction between aSyn and microtubule elements have been shown in our own studies [22,52] and other studies (reviewed in [53,54]). Moreover, the study of Cartelli et al. suggested that aSyn may function as a dynamase that modulates the microtubule organization [55]. Interestingly, their data demonstrated that PD-linked aSyn variants can cause tubulin aggregation but not organized polymerization in a cell-free model system. This effect of aggregated aSyn on tubulin disorganization was supported by our observations in *SNCA^Dupl^* neurons [22]. In total, our data support the hypothesis that perturbation of aSyn subcellular shuttling kinetics in PD might promote the accumulation of nucleus-derived aSyn, probably in aggregated forms, in neurites, leading to neuritic degeneration via the interference with microtubule organization. Further studies are needed to elucidate the toxicity of aSyn species moving from the nucleus to neurites, as well as the significance and regulatory effects of the translocation on microtubule organization in health and disease. The better understanding of the dynamic intracellular distribution of aSyn is important to identify novel pathways in neurodegeneration and help us to identify new therapeutic interventions altering the disease course in PD.

## 5. Conclusions

The present study describes distinct aSyn species, which exhibit a characteristic nuclear–somatic–neuritic shuttling during neuronal differentiation. This particular aSyn translocation pattern was only recognized by a specific anti-aSyn Nu-aSyn-C antibody, which has a particular high immunoaffinity to aSyn in the nucleus of proliferating cells, and has a high binding capacity to aggregated aSyn. Using additional complementary aSyn antibodies, we further revealed that nuclear aSyn from proliferating cells has a higher phosphorylation and assembly level. Our data suggest that a translocation of distinct assembled aSyn species in developing neurites could be physiologically required to maintain neuronal homeostasis. *SNCA* gene duplication disrupts this homeostasis and induces a shift of Nu-aSyn-C-positive species in mDANs towards the neurites. The increased Nu-aSyn-C-positive aSyn load along the neurites may explain the neuritic phenotypes in *SNCA^Dupl^* mDANs described in our previous studies.

## Data Availability

The data presented in this study are available in this article and the supplementary materials.

## Supplementary Materials

The following supporting information can be downloaded at: https://www.mdpi.com/article/10.3390/biom12081108/s1, Figure S1: Immunocytochemical detection of aSyn using the Nu-aSyn-C antibody in cortical tissue of WT and aSyn KO mice. Figure S2: Western blot analysis of H4 cells using Nu-aSyn-C and Syn1 antibodies. Figure S3: Schematic summary of midbrain dopaminergic neuronal differentiation protocols for LUHMES cells and hiPSCs. Figure S4: Subcellular localization of aSyn detected with the Nu-aSyn-C antibody in primary rat hippocampal neurons. Figure S5: Distribution of Nu-aSyn-C-positive aSyn species in hiPSC-derived mDANs from healthy controls and a *SNCA^Dupl^* patient [56].
