# Supplementary material for: Translocation of Distinct Alpha Synuclein Species from the Nucleus to Neuronal Processes during Neuronal Differentiation
Source: Biomolecules. 2022 Aug 12;12(8):1108. doi: 10.3390/biom12081108 (PMC9406079; doi:10.3390/biom12081108)
Supplement: Supplementary file 1 [file biomolecules-12-01108-s001.zip › biomolecules-1809737-supplementary.pdf]

Supplementary Materials

Translocation of distinct alpha synuclein species from the nucleus to neuronal processes during neuronal differentiation

Katharina Pieger[1], Verena Schmitt[2], Carina Gauer[2], Nadja Gießl[1], Iryna Prots[3], Beate Winner[3], Jürgen Winkler[2], Johann Helmut Brandstätter[1],Wei Xiang[2*]

[1] Department of Biology, Animal Physiology/Neurobiology, Friedrich-Alexander-Universität Erlangen-Nürnberg, 91058 Erlangen, Germany
[2] Department of Molecular Neurology, University Hospital Erlangen, Friedrich-Alexander-Universität Erlangen-Nürnberg, 91054 Erlangen, Germany
[3] Department of Stem Cell Biology, University Hospital Erlangen, Friedrich-Alexander-Universität Erlangen-Nürnberg, 91054 Erlangen, Germany
* Correspondence: Wei Xiang, PhD, Department of Molecular Neurology, University Hospital Erlangen, Friederich-Alexander-Universität Erlangen-Nürnberg, Schwabachanlage 6, D-91054 Erlangen, Germany, wei.xiang@fau.de; Tel.: +49 9131 85 44676

Figure S1. Immunocytochemical detection of aSyn using the Nu-aSyn-C antibody in cortical tissue of WT and aSyn KO mice.

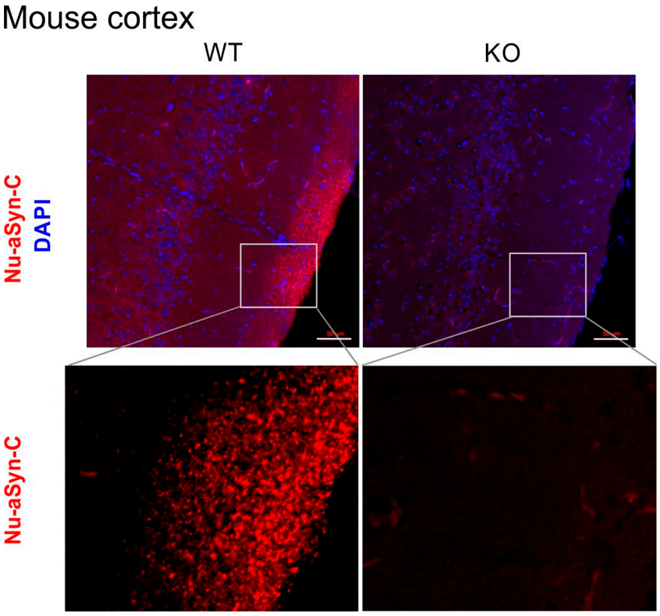

Immunofluorescent staining of aSyn with the Nu-aSyn-C antibody and visualization of cellular nuclei with DAPI (blue) were performed on slices of murine cortical tissue. aSyn is detected by the Nu-aSyn-C antibody only in WT mice. Scale bars: 50 µm.

Figure S2. Western blot analysis of H4 cells using Nu-aSyn-C and Syn1 antibodies.

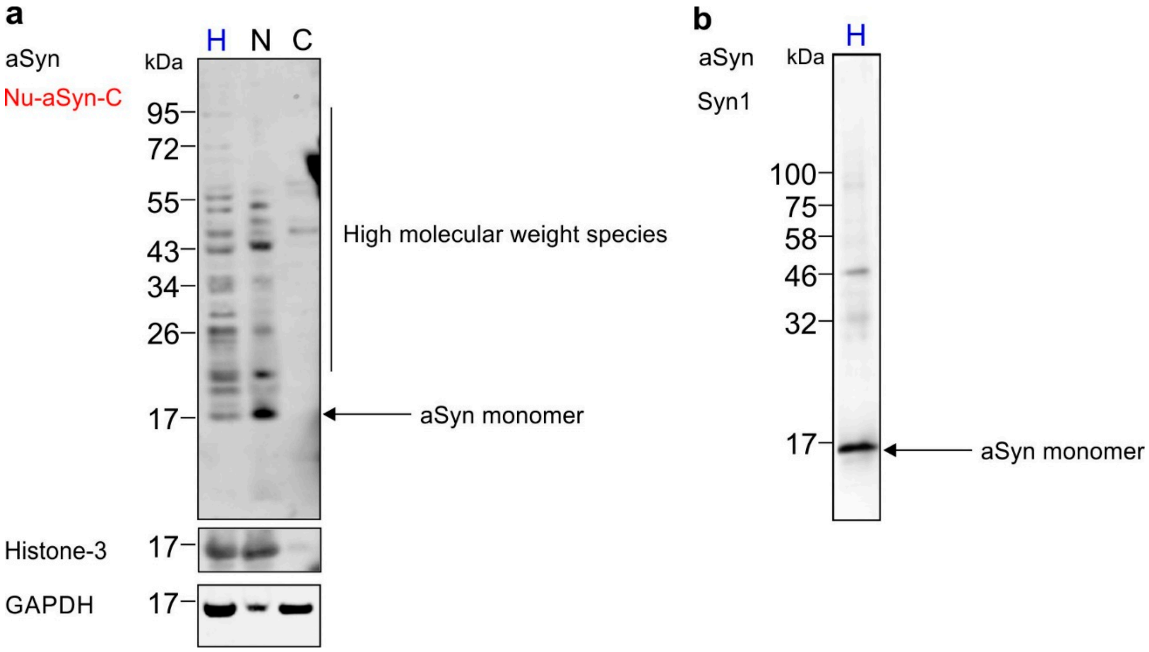

aSyn in the whole cell homogenate (H) of H4 cells was probed by using Nu-aSyn-C (a) and Syn1 (b) antibodies. In addition, nuclear (N) and cytosolic (C) fractions were analyzed by using Nu-aSyn-C. Due the low expression level in H4 cells, aSyn in H4 cells probed by the Syn1 antibody (in b) was detected by using a HRP-conjugated anti-mouse secondary antibody and the high sensitive SuperSignal™ West Femto Substrate (ThermoFisher Scientific). GAPDH and histone 3 serve as protein markers of the cytosol and the nucleus, respectively.

Figure S3. Schematic summary of midbrain dopaminergic neuronal differentiation protocols for LUHMES cells and hiPSCs.

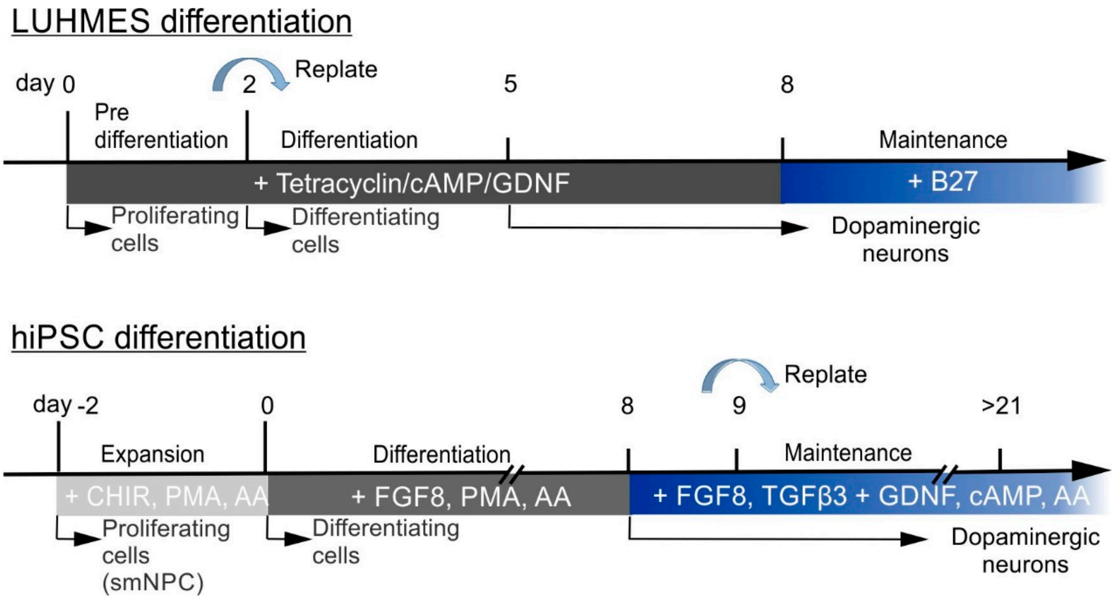

For more details please refer to the section "Materials and Methods" of the manuscript.

Figure S4. Subcellular localization of aSyn detected with the Nu-aSyn-C antibody in primary rat hippocampal neurons.

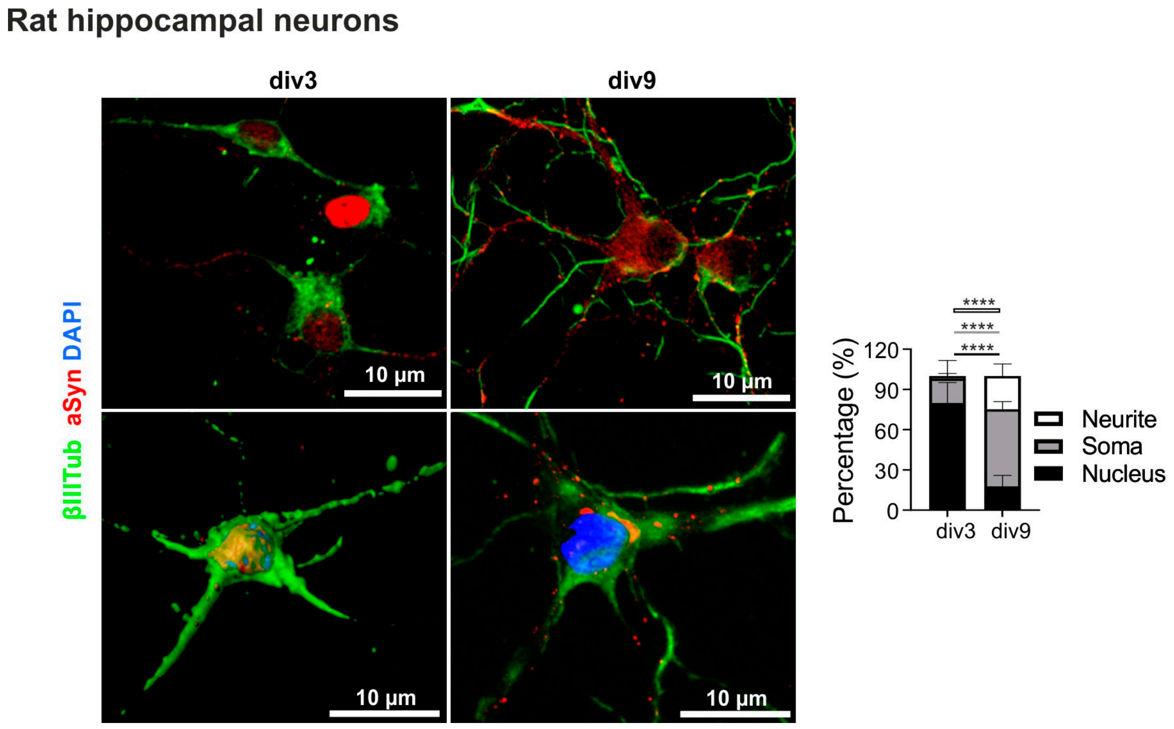

Primary rat hippocampal neurons were prepared as described previously [1]. The primary cells were maintained in serum-free neurobasal medium containing B27 supplement (Thermo

Fisher Scientific) and kept at 37°C in 5% CO2 for 3 – 9 d*iv* (days *in vitro*). ICC images of aSyn (red) were taken after 3 and 9 d*iv*, respectively (upper panel) and the fluorescent signals of representative neurons were reconstructed in 3D (lower panel). Cellular nuclei and neurites were visualized with DAPI (blue) and βIIITub-staining (green), respectively. Bar diagram comparing the relative percentage distribution of aSyn within the neuritic, somatic and nuclear cell compartments in neurons of d*iv*3 (n = 10) and d*iv*9 (n = 11). Statistics: Two way ANOVA, Tukey's multiple comparisons test ****p<0.0001.

Figure S5. Distribution of Nu-aSyn-C-positive aSyn species in hiPSC-derived mDANs from healthy controls and a *SNCA*^{Dupl} patient.

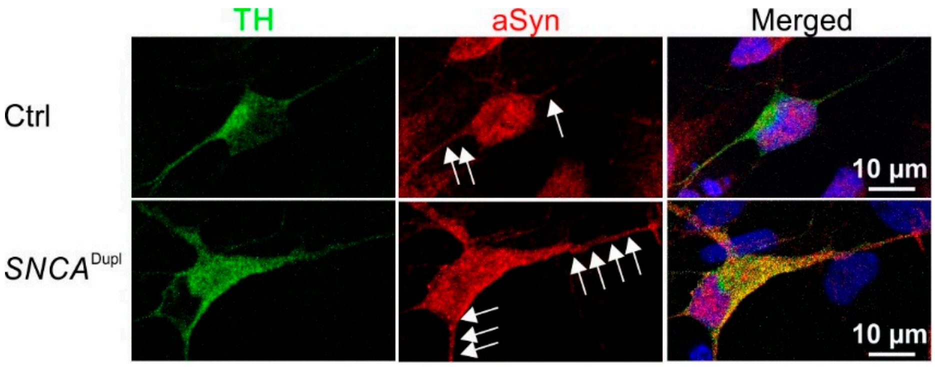

Representative immunofluorescence images of mDANs differentiated for 21 days labelled with Nu-aSyn-C, anti-TH and DAPI. Corresponding βIIItub staining is shown in Figure 6.

Reference:

1. Kaech S.. Banker G. Culturing hippocampal neurons. *Nat Protoc*. **2006**;1(5):2406-15.
